# The Impact of the Mediterranean Diet on Telomere Biology: Implications for Disease Management—A Narrative Review

**DOI:** 10.3390/nu16152525

**Published:** 2024-08-02

**Authors:** Stella Baliou, Petros Ioannou, Miruna-Maria Apetroaei, Elena Vakonaki, Persefoni Fragkiadaki, Evangelos Kirithras, Manolis N. Tzatzarakis, Andreea Letitia Arsene, Anca Oana Docea, Aristides Tsatsakis

**Affiliations:** 1 Laboratory of Toxicology, Medical School, University of Crete, 71003 Heraklion, Greece; s.baliou@med.uoc.gr (S.B.); evakonaki@gmail.com (E.V.); persefoni.f@gmail.com (P.F.); vaggelis@libero.it (E.K.); tzatzarakis@med.uoc.gr (M.N.T.); tsatsaka@uoc.gr (A.T.); 2 Lifeplus S.A., Science & Technological Park of Crete, C Building, Vassilika Vouton, 70013 Heraklion, Greece; 3 School of Medicine, University of Crete, 71003 Heraklion, Greece; 4 Faculty of Pharmacy, Carol Davila University of Medicine and Pharmacy, 6, Traian Vuia Street, 020956 Bucharest, Romania; miruna-maria.apetroaei@rez.umfcd.ro (M.-M.A.); andreea.arsene@umfcd.ro (A.L.A.); 5 Department of Toxicology, University of Medicine and Pharmacy of Craiova, 200349 Craiova, Romania; 6 Department of Toxicology, Faculty of Pharmacy, University of Medicine and Pharmacy, Petru Rares, 200349 Craiova, Romania

**Keywords:** telomere length, Mediterranean diet, polyphenols, vitamins, minerals, fatty acids, antioxidant, anti-inflammatory, diseases, autoimmunity

## Abstract

Introduction: Telomeres are nucleoprotein complexes at the ends of chromosomes that are under the control of genetic and environmental triggers. Accelerated telomere shortening is causally implicated in the increasing incidence of diseases. The Mediterranean diet has recently been identified as one that confers protection against diseases. This review aimed to identify the effect of each component of the Mediterranean diet on telomere length dynamics, highlighting the underlying molecular mechanisms. Methods: PubMed was searched to identify relevant studies to extract data for conducting a narrative review. Results: The Mediterranean diet alleviates clinical manifestations in many diseases. Focusing on autoimmune diseases, the Mediterranean diet can be protective by preventing inflammation, mitochondrial malfunction, and abnormal telomerase activity. Also, each Mediterranean diet constituent seems to attenuate aging through the sustenance or elongation of telomere length, providing insights into the underlying molecular mechanisms. Polyphenols, vitamins, minerals, and fatty acids seem to be essential in telomere homeostasis, since they inhibit inflammatory responses, DNA damage, oxidative stress, mitochondrial malfunction, and cell death and induce telomerase activation. Conclusions: The Mediterranean diet is beneficial for maintaining telomere dynamics and alleviating age-related illnesses. This review provides a comprehensive overview of cross-sectional, observational, and randomized controlled trials regarding the beneficial impact of every constituent in the Mediterranean diet on telomere length and chronic disease management.

## 1. Introduction

Aging is characterized by genome instability, senescence, telomere shortening, mitochondrial malfunction, stem cell exhaustion, loss of proteostasis, altered nutrient and cellular communication, and epigenetic modifications [1]. Biological aging has been acknowledged as a more accurate measure of disease-specific risk than chronological aging [1].

Among all the aging hallmarks, telomere dysfunction is the main characteristic of aging that leads to the induction or exacerbation of other aging markers [2]. Telomeres have garnered much attention in the past decades due to their role as primary gatekeepers of genome stability [1]. Because the 3′ ends of telomeric deoxyribonucleic acid (DNA) cannot be fully replicated, telomeres become shortened throughout each cell cycle because of insufficient replication of telomeric DNA, leading to either senescence or cell death. After each cell replication cycle, shelterin protein complex components may be displaced from telomeres, disrupting their t-loop conformation. In this manner, the increasing loss of telomere repeats with each cell division causes the subsequent activation of a DNA damage response (DDR), since telomeres are recognized as exposed sites for recruiting DNA repair machinery [1]. Indeed, prolonged DDR is a primary aging mechanism that triggers p53 activation and sustains permanent cell cycle arrest, thus contributing to senescence [3]. 

Cellular senescence is distinguished by alterations in chromatin, gene expression, organelles, and cell shape, in addition to irreversible cell cycle arrest [1]. There is a growing body of research supporting that telomere dysfunction is the primary event driving senescence. Τelomere dysfunction can arise after chronic inflammation [4] and mitochondrial dysfunction [5]. Indeed, senescence is characterized by the impairment of mitochondrial function and inflammation, which emerge through the mitochondrial dysfunction-associated senescent response (MiDAS) and the senescence-associated secretory phenotype (SASP) [4,5]. According to the mitochondrial free radical theory of aging, as people age, their mitochondria start to undergo defects, resulting in an increase in the generation of reactive oxygen species (ROS), which exacerbates damage to mitochondria and triggers additional destruction of cells throughout the body [5]. Based on this, the mitochondrial free radical theory of aging was proposed to highlight the significant role of mitochondrial dysfunction during aging [6].

Additionally, there is a close interplay between mitochondrial malfunction and telomere dysfunction. On the one hand, mitochondrial oxidative stress is the primary driving force that causes DNA and telomere damage, leading to genomic instability [7,8]. In particular, telomeres are susceptible to oxidative damage since they are rich in the G context, making them more prone to oxidative DNA lesions than the rest of the genome [9]. In particular, ROS account for the increased incidence of 8-oxoguanine (8-oxo-G) lesions in telomeres [8], which cannot be removed by the corresponding enzyme 8-oxoguanine glycosylase (OGG1) [10]. The increased vulnerability of telomeric DNA sequences to oxidative damage is also attributed to the more cumulative binding of iron (Fe^2+^) on telomeres compared to other genome sequences, thereby perpetuating the secretion of hydroxyl radicals through Fenton reactions [11]. As a result, there is substantial evidence that oxidative stress accounts for telomere damage, accelerating telomere shortening and progression of age-related disorders [12]. In addition, the aging process is exacerbated when the cell does not repair damage to telomeric DNA sequences, exacerbating the proinflammatory SASP [7]. On the other hand, there is a positive feedforward loop in which telomere loss contributes to mitochondrial dysfunction. Indeed, telomere shortening has been reported to induce a sequence of events, with reduced mitochondrial membrane hyperpolarization being the initial step after increased telomere response to damage. In particular, p53-mediated telomere damage has been reported to cause disrupted mitochondrial respiration, since activated p53 tumor suppressor is recruited at the promoters of peroxisome proliferator-activated receptor gamma, coactivator 1 alpha and beta (*PGC1A* and *PGC1B*), hindering the expression of genes involved in mitochondrial biogenesis [13].

Lastly, chaperone function, protein stability, and folding are also significantly disturbed in aging [14]. Apart from this, all cells can be affected by a range of epigenetic changes at different stages of life [15]. Chromosome reconstruction, post-translational modifications of histones, and variations in DNA methylation patterns are examples of epigenetic modifications. Such age-associated epigenetic changes include increased histone H4K16 acetylation, H4K20 trimethylation, and H3K4 trimethylation, as well as decreased H3K9 methylation and H3K27 trimethylation [16].

Even though there are studies that have reviewed the effect of the MD on telomere length, the constituents of the MD and the associated mechanisms affecting telomere length dynamics have not been adequately described. In this narrative review, we aimed to identify the effect of each MD component on telomere length dynamics, giving particular emphasis to the underlying molecular mechanisms in an attempt to provide insights into their protective effects against disease.

## 2. Search Strategy

A search of the PubMed database for relevant articles published until the end of May 2024 was conducted to examine the effects of MD constituents, specifically polyphenols, vitamins, and minerals, on aging and autoimmune diseases and on telomere length. “(Mediterranean diet OR polyphenols OR vitamins OR minerals OR fatty acids) AND telomere length AND diseases” was the search query. Many relevant reviews and original research articles offered information on the topic, and they were assessed and incorporated into this narrative review in a liberal, non-systematic manner. After screening the studies, the two investigators (SB and PI) took out and combined the evidence. The relevant systematic and narrative review references were searched to identify other pertinent articles.

## 3. The Beneficial Effects of the MD on Diseases

In humans, telomere shortening has been linked to a wide range of illnesses, including mental problems, cardiovascular disease (CVD), cancer, liver cirrhosis, diabetes mellitus, osteoporosis, and osteoarthritis [17,18,19,20,21,22,23,24]. From a molecular perspective, the appearance of critically short telomeres has been reported to coincide with chromosomal degradation, end-to-end fusion, and deficient recombination, contributing to age-associated disorders [3]. Epidemiological evidence has highlighted the increased susceptibility to mortality owing to telomere shortening [25]. 

Since oxidative stress serves as a drug target for inhibiting cell senescence, antioxidant substances included in the MD can be classified as senolytics, which preferentially destroy senescent cells, or senomorphics, which alter the senescence phenotype [26]. On the one hand, senolytics function through suppression of the BCL-2 antiapoptotic family, negative modulation of the phosphatidylinositol 3′-kinase (PI3K)/Akt pathway, and FOXO regulation [26]. On the other hand, senomorphics downregulate SASP expression, slowing or reversing senescence [26]. A variety of food elements in the MD have shown senolytic effects. Nuts and some vegetables, for instance, have been reported to halt the growth of senescent cells [27]. 

The MD, as a senolytic dietary strategy, is among the healthiest diets worldwide, and it has received a lot of interest throughout the last several decades. High intakes of vegetables, legumes, fruits, nuts, and cereals are the main characteristics of the traditional MD. Additionally, high consumption of olive oil and low intake of saturated lipids, moderately high intake of fish, low to moderate consumption of dairy products, low intake of meat and poultry, and regular but moderate intake of ethanol—mostly in the form of wine and usually during meals—are additional features of the standard MD [28,29]. Notably, combining a healthy diet and exercise can attenuate the progression of age-related disorders caused by telomere shortening [26,27]. 

Since the 1960s, the MD has gained attention as a suitable plant-based diet that lowers the possibility of several age-related illnesses, such as neurodegeneration, cancer, metabolic syndrome (MetS), diabetes, and CVD [30,31,32,33]. In this regard, a meta-analysis has highlighted the clinical importance of the MD dietary strategy in attenuating many chronic disorders like diabetes, cancer, cardiac events, neural disorders, and general mortality [34]. Accordingly, a recent systematic review has demonstrated that an MD dietary strategy can attenuate the risk of obesity, cardiac diseases, and mental disorders, thus mitigating telomere shortening and supporting longevity [35].

**Figure 1:**
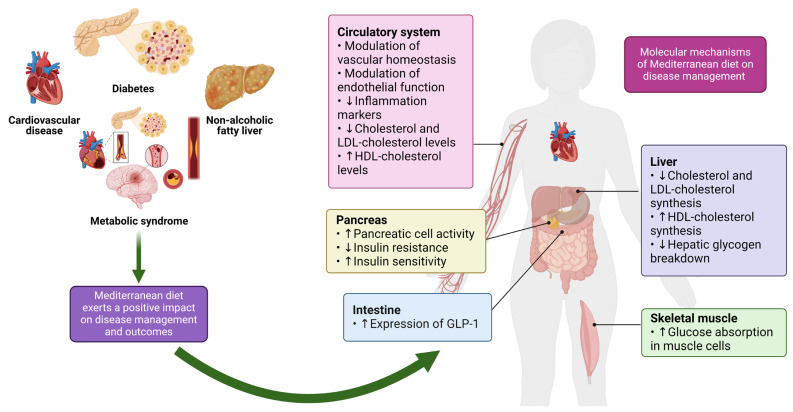
**The Mediterranean diet exerts a protective effect on organ systems.** The Mediterranean diet ameliorates the function of the circulatory system, the pancreas, the intestine, the liver, and skeletal muscle. Abbreviations: GLP-1: glycagon-like-peptide-1; LDL: low-density lipoprotein; HDL: high-density lipoprotein. (Created with BioRender.com.)

A vast body of research has highlighted the beneficial effects of the MD dietary strategy on several organs, including the circulatory system, the pancreas, the liver, the intestine, and skeletal muscle [36] (Figure 1).

Concerning cardiac events, an important randomized nutritional intervention study showed the positive effect of the MD dietary strategy on telomere length in CVD-risk individuals [37]. It was proved there was a close relationship between telomere length elongation in women prone to CVD events and following the MD dietary strategy [37]. Accordingly, a large meta-analysis showed that fruits, vegetables, beans/legumes, nuts/seeds, whole grains, fish, yogurt, fiber, and omega-3 fatty acids from fish were linked to a lower CVD risk [38]. In contrast, processed meats, unprocessed red meats, sugar-sweetened beverages, and sodium were related to higher CVD risk [38]. In another meta-analysis, it was proved that high saturated fatty acid (SFA) intake through either sugar-sweetened beverages or processed meat intake contributed to increased CVD incidence, whereas a healthy diet composed of fruits, legumes, and fish reduced CVD risk [39]. To confirm this, many observational studies have shown the link between SFA consumption and increased CVD incidence [40,41,42].

In a clinical setting, MD exerted a beneficial effect by attenuating clinical signs of cardiac diseases. In the Oslo Diet-Heart RCT, individuals with a history of myocardial infarction were recruited, and the effects of a diet low in SFAs and cholesterol and a diet high in PUFAs were compared [43]. After five years of the trial, the study’s results showed that the participants who followed the low-SFA diet had a reduced risk of developing CVD events and subsequent mortality [43]. In addition, the Finnish Mental Hospital elucidated the impact of a diet enriched in PUFAs and low in SFAs on CVD development, showing that the development of CVD was reduced in participants who followed a low-SFA, high-PUFA diet [44]. Interestingly, the PREvención con DIeta MEDiterránea (PREDIMED) RCT provided the insight that higher consumption of monounsaturated fatty acids (MUFAs) and polyunsaturated fatty acids (PUFAs), which are included in the MD, decreased susceptibility to CVD progression and death [45]. In contrast, high SFA intake exerted a detrimental effect on cardiac function, increasing the risk of CVD development [45]. Accordingly, the PREDIMED-Plus RCT provided convincing evidence that MetS women had attenuated telomere shortening after a three-year lifestyle adjustment comprising the MD plan and exercise [46]. In addition, a cohort from the Moli-sani study showed that MD was beneficial in preventing CVD risk due to MUFA abundance [47]. In particular, the increased ratio of MUFAs was effective in minimizing the risk factors for CVD development, including lipid profile and inflammatory markers, compared to SFA consumption [47].

Endothelial dysfunction is also a key stage in the development of atherosclerosis, and it determines the likelihood of CVD recurrence. In this regard, the CORDIOPREV prospective single-blind RCT compared the impact of two healthy dietary patterns, namely, low-fat and MD dietary plans, on the incidence of CVD in patients with coronary heart disease (CHD) [48]. It was proved that the low-fat diet was not as effective as the MD in reducing significant CVD events [48]. Similarly, the MD seems to normalize vascular homeostasis and regulate endothelial function compared to a low-fat diet [49].

Regarding the benefits of the MD plan in metabolic diseases, the mechanisms behind the MD’s protective action are based on the attenuation of insulin resistance and inflammation [50]. More specifically, a diet high in MUFAs and low in SFAs has been linked to improved pancreatic cell activity and enhanced insulin sensitivity [51]. Indeed, it has been demonstrated that the unsaturated fatty acids in olive oil increase the expression of glucagon-like-peptide-1 (GLP1) [52], since they suppress glycogen secretion while increasing insulin secretion [51]. Constituents of the MD with health-inducing benefits against diabetes are polyphenols that enhance glucose absorption from muscle cells, hinder hepatic glycogen breakdown, and prevent the inflammation-associated deficiency of pancreatic cells [53]. Notably, the polyphenol quercetin can act similarly to the classically used antidiabetic drug metformin, attenuating mitochondrial dysfunction [54]. In addition to the above, the management of non-alcoholic fatty liver disease (NAFLD) has been reported to be accomplished through MD components due to their lipid-lowering, antioxidant, and anti-inflammatory properties [50]. To support this, an RCT has highlighted the beneficial effect of the MD strategy in alleviating hepatic steatosis [55]. Accordingly, a low-glycemic index Mediterranean diet (LGIMD) has been proved to be effective in attenuating [high-density lipoprotein cholesterol (HDL-C)-low-density lipoprotein cholesterol (LDL-C)] as a percentage of total cholesterol, termed fasting remnant cholesterol (REM-C), after three or six months in MetS patients [56]. The results mentioned above were considered given that MetS and NAFLD patients have an increased risk of CVD due to elevated fasting remnant cholesterol (REM-C) levels for months [56]. 

Regarding the association of the MD dietary plan and cancer, consuming whole grains and other high-fiber foods may minimize the risk of developing several cancers, including colorectal, lung, stomach, breast, esophageal, and oral cancers [57]. According to a recent systematic review, the adoption of plant-based diets, especially the MD option, is associated with significant improvements in tumor growth restriction, recurrence-free status, and diminishing the development of a variety of cancers [58]. Relatedly, another systematic review of observational studies suggested that higher adherence to the MD dietary option can alleviate the risk of colorectal adenomas (CRAs) [59].

Additionally, many studies have highlighted the contribution of the MD dietary option to ameliorating autoimmune disease pathogenesis [60]. 

For example, a prospective interventional study on children with type 1 diabetes (T1D) showed the beneficial effect of the MD strategy. The authors reported that the MD plan presents significant effects in terms of blood pressure and improvement in MUFA intake [61]. The relationship between the MD strategy and T1D pathogenesis was also studied in adolescents, suggesting that the MD effectively improves glycemic status in adolescents with T1D [62]. In this context, another recent study proved that the MD plan minimizes CVD events and significantly enhances glycemic management in patients with autoimmune diabetes [63]. 

From a molecular perspective, the MD dietary option favors the immune system, microbiota, and redox balance through its immunomodulatory and antioxidant action [60]. In addition, many experimental models of autoimmune thyroiditis have shown that one of the main components of MD, extra virgin olive oil, reduces the risk and complications associated with this disease [60]. It has been hypothesized that oleic acid and hydroxytyrosol inhibit oxidative stress and inflammation by enhancing the nuclear factor erythroid-2-related factor/heme-oxygenase 1 (Nrf2/HO-1) signaling pathway and inhibiting the mitogen-activated protein kinase/nuclear factor-kappa B (MAPK/NF-κB) signaling pathway [60]. In line with the above, the most prevalent autoimmune illness, Hashimoto’s thyroiditis (HT), is the primary cause of hypothyroidism, a condition in which the thyroid gland’s function is compromised by lymphocyte infiltration. In this autoimmune thyroid disease, the MD dietary strategy seems beneficial [64]. In particular, a diet low in animal products and high in vitamins and minerals may have a prophylactic impact on the incidence of Hashimoto’s thyroiditis [64]. 

## 4. The Effect of the MD on Telomere Length Dynamics

Telomere length is considered a strong biological age indicator, and telomere dynamics can be affected by genetics, environmental influences, socioeconomic status, and lifestyle decisions, including food intake and physical activity [65].

On the one hand, it has been reported that red and processed meat, sweetened beverages that contain sodium, and white bread consumption have all been associated with increased telomere shortening in the human setting, since they initiate DDR due to oxidative stress or inflammation [66,67]. A systematic review has provided convincing evidence that high adherence to the MD dietary strategy is positively associated with telomere length maintenance or elongation [68]. 

In specific demographic subgroups, leukocyte telomere length and MD adherence seem to have a favorable relationship [11,53]. For example, a group of nurses from southern Italy and nurses between the ages of 30 and 55 showed telomere length elongation depending on their adherence to the MD dietary plan [69]. After controlling for relevant unknown variables, longer telomeres were observed in women who adhered more strictly to the MD dietary plan [69]. The researchers revealed that even modest dietary adjustments could significantly impact telomere length [69]. In parallel with this, they found no evidence of statistical significance in the association of Western dietary habits with telomere length [69].

Furthermore, two Mediterranean populations—one on the island of Ikaria in Greece and the other in the Sicani Mountains of Sicily, Italy—showed that the two populations’ longevity was accomplished by following the MD dietary plan [70]. Elderly men living in Greece displayed longer telomeres and more antioxidant responses than elderly Dutch men who lived in a more stressful urban environment and consumed a diet lower in antioxidant elements, implying a causal connection between aging-related telomere length shortening and elevated oxidative stress [71]. In another cohort study of elderly Spanish individuals, telomere length elongation was observed at a greater extent when a high-quality diet was followed. Interestingly, individuals who were positively assessed using the Prime Diet Quality Score (PDQS), the Mediterranean Diet Adherence Screener (MEDAS), and Dietary Approaches to Stop Hypertension (DASH) had a lower probability of having short telomeres than those who followed a classical diet [72]. Consistent with this, Trichopoulou et al. invented the MD score to show the extent of adherence to the MD dietary plan [28]. In this regard, Italians also showed telomere length elongation according to their degree of compliance with healthy dietary habits [73]. The aforementioned examples support the idea that the populations’ strict compliance with the MD was tightly related to longevity.

Several meta-analyses have highlighted the great impact of the MD nutritional option on telomere length dynamics (Table 1). The systematic review results initially showed that fruits and vegetables impact aging favorably, contributing to telomere length elongation [74]. In contrast, telomere shortening occurred due to the presence of specific dietary groups, such as cereals, processed meat, and sugar-sweetened beverages [74]. As a result, a healthy diet can hinder telomere shortening, whereas a Western diet consisting of pickled vegetables, rice, and red meat can accelerate telomere shortening [74]. Another systematic review of cross-sectional studies showed that telomere maintenance can be accomplished through adherence to the MD dietary option [46]. Consistent with this, a recent meta-analysis proved that telomere length elongation is correlated with higher MD compliance [75]. Notably, the beneficial effects of the MD were observed to be more pronounced in women. Accordingly, Meinilä et al. showed the attenuating effect of the MD strategy on telomere shortening in Dutch women in a study with a follow-up period of 10 years [76]. 

**Figure 2:**
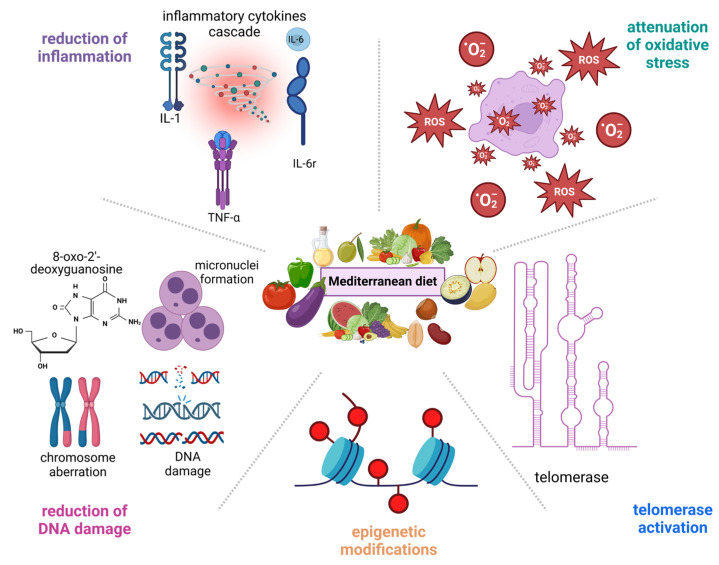
**The molecular mechanisms underlying the protection by the Mediterranean diet.** The benefits of the Mediterranean diet are ascribed to reductions in DNA damage, inflammation and oxidative stress, telomerase activation, and epigenetic modifications. Abbreviations: TNF−α: tumor necrosis factor−α; il−6: interleukin6; ROS: reactive oxygen species; O_2_^−^: superoxide anions. (Created with BioRender.com.)

From a molecular perspective, the main mechanisms of action underlying the MD’s protective properties are based on its anti-inflammatory and antioxidative components. The beneficial effect of MD food components is ascribed to reductions in oxidative stress, inflammation, DNA damage, and telomerase induction (Figure 2) [73,77]. 

**Table 1:** The effect of the Mediterranean diet on telomere length dynamics.

Effect of the MD on TL	Number of Individuals	Method	Reference
Greater adherence to the MD is associated with longer TL	32,825 women provided blood samples in 1989–1990	qPCR	[69]
Significantly longer TLs in Greek elderly men compared to Dutch counterparts due to MD consumption	143 elderly Dutch men (mean age: 83.9 years) and 109 Greek elderly men (mean age: 84.6 years)	qPCR	[71]
Positive effect of a 5-year MD intervention on TL	521 people (55–80 years, 55% women)	qPCR	[78]
Lower TL shortening with MD	865 older Spanish people (>55 years old)	qPCR	[72]
Reduced TL shortening in women in a study with a 10-year follow-up	Dutch participants	qPCR	[76]

Abbreviations: MD: Mediterranean diet; TL: telomere length; qPCR: quantitative polymerase chain reaction. The table is not exhaustive of all available studies.

Initially, the MD nutritional option has been reported to prevent telomere shortening due to its anti-inflammatory properties and epigenetic modifications [27]. Several studies have supported the anti-inflammatory nature of the MD nutritional option. The Prevention with Mediterranean Diet study revealed that an MD dietary strategy supplemented with extra virgin olive oil (EVOO) and/or nuts reduced proinflammatory mediators like tumor necrosis factor-alpha (TNF-α), interleukin-6 (IL-6), IL-12, and interferon-gamma (IFN-γ), as well as oxidative stress biomarkers like F2-isoprostanes. In the PREDIMED RCT, participants experienced increased telomere length after a 5-year MD intervention [78]. In individuals 55 years of age or older, a positive relationship between telomere shortening and increased inflammation was substantiated [79]. Similarly, long telomeres and low inflammatory responses were observed in populations of centenarians (100–104 years old) and elderly individuals (85–99 years old). Compared to age-matched controls, the progeny of centenarians displayed equal telomere length elongation and lower basal inflammatory activity [80]. According to the results of that study, low inflammation was considered to have a prognostic value for predicting healthy aging [80]. In a molecular setting, telomere shortening and the onset of senescence seem to be the result of hyperactivity of the transcriptional factor nuclear factor-kappa beta (NF-kB) and increased expression of pro-inflammatory cytokines, including TNF-α, IL-6, and IFN-γ, in macrophages [81]. 

The MD dietary strategy is known for its antioxidant properties and to prevent telomere shortening [82]. Antioxidants can be found in a variety of foods, including broccoli, sprouts, red grapes, tomatoes, olive fruit, salmon, tuna, mackerel, herring, halibut, catfish, anchovies, grouper, flax seeds, flounder, chia seeds, kiwi, black raspberries, sesame seeds, and lingonberries, which ensure telomere stability against oxidative damage [66]. For example, the consumption of seeds or products derived from them, such as coffee, almonds, and legumes, had a positive effect on leukocyte telomere length in Korean adults [83]. 

In addition, the MD seems to be beneficial in reducing telomere shortening by preventing DNA damage. For example, the effectiveness of specific micronutrients for acquiring DNA integrity has been reported. DNA damage measurements are usually accomplished through determination of micronuclei (MN) frequencies, chromosome aberrations (Cas), and increased appearance of 8-oxo-2′-deoxyguanosine (8-oxo-dG) lesions [84].

Another mechanism underlying the protective effects of specific foods against telomere shortening is based on the upregulation of telomerase action. For example, Ornish et al. evaluated the effect of a healthy lifestyle comprising increased physical activity and stress management on aging, suggesting their positive effect on telomere length dynamics [85]. Indeed, the participants followed a plant-based diet rich in fruits, vegetables, whole grains, and legumes and low in fat (around 10% of calories) and refined carbs. Fish oil, vitamin C, vitamin E, selenium, and soy products were also included in the diet as supplements [85]. The results proved that a healthy lifestyle benefits aging, since it ameliorated telomerase activity in the peripheral blood mononuclear cells (PBMCs) of the participants over three months [85]. In individuals following a healthy lifestyle, PBMCs demonstrated a substantial 29% increase in telomerase activity compared to the baseline, as proved by the telomerase repeated amplification protocol (TRAP) assay [85]. Accordingly, higher adherence to the MD dietary strategy was associated with higher telomerase activity and, consequently, longer telomeres [86]. Regardless of other confounders, it was proved that MD induces telomerase action, leading to telomere length elongation, since telomerase is negatively affected by inflammation and oxidative stress [86].

## 5. The Effects of MD Constituents on Telomere Length Dynamics

**Figure 3:**
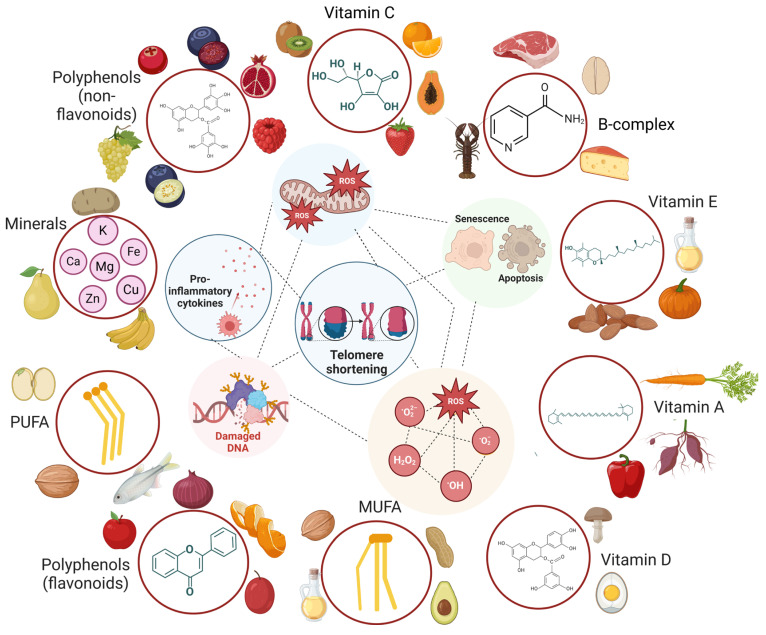
**The protective effects of constituents included in the Mediterranean diet**. The main components of the Mediterranean diet are polyphenols, polyunsaturated fatty acids, monounsaturated fatty acids, vitamins, and minerals. They can inhibit telomere shortening by attenuating inflammation, oxidative stress, DNA damage, mitochondrial dysfunction, and cell death. Abbreviations: ROS: reactive oxygen species; PUFAs: polyunsaturated fatty acids; MUFAs: monounsaturated fatty acids; Ca: calcium; K: potassium; Fe: iron; Mg: magnesium; Zn: zinc; Cu: copper. (Created with BioRender.com.)

A growing body of research delineates the distinct or synergistic effects mediated by different food components included in the MD dietary plan. Better adherence to the MD dietary plan is related to reduced telomere shortening to a greater extent [87]. In particular, some micronutrients like vitamins (including folate and vitamins A, B12, C, D, and E), minerals (such as magnesium, zinc, and iron), and other dietary components (such as fatty acids and polyphenols) have been revealed to exert a significant impact on sustaining or elongating telomere length through several mechanisms [88] (Figure 3). Importantly, a systematic review of randomized controlled interventions and uncontrolled longitudinal intervention studies provides insights into the role of micronutrients in delaying aging. In particular, folate, vitamin B_12_, and zinc seem to be involved in DNA metabolism and repair. In contrast, vitamins A, C, and E and zinc neutralize the accrual of DNA damage through the inhibition of oxidative stress and inflammation [84].

### 5.1. The Effects of Polyphenols on Telomere Length Dynamics

In response to a stressful environment, plants synthesize polyphenols, the greatest secondary metabolites with no energy value [89]. These are essential for human health [90]. Based on their chemical structure, polyphenols have been classified into different classes. These classes, flavonoids and non-flavonoids, are further divided into flavonols, flavanols, anthocyanins, flavones, procyanidins, stilbenes, phenolic acids, and tannins based on the number of hydroxyls in the molecule and the type and position of the other substituents [91]. Plant foods such as fruit, vegetables, dried legumes, olives, grains, tea, chocolate, and wine are the primary sources of dietary polyphenols [92]. In particular, polyphenols are contained in the following foods: quercetin in onions and grapes; tannins in tea and nuts; lignin in whole grains and nuts; pro-anthocyanidins in grapes, pine bark, and cocoa; naringenin/hesperidin in citrus fruits; resveratrol in wines; catechins in grapes, wine, and green tea; and anthocyanins/anthocyanidins in vibrantly colored fruits and vegetables like berries [93].

Numerous polyphenolic compounds operate as powerful antioxidants in vivo and in vitro, shielding against oxidative stress [52]. There is compelling evidence that these phytochemicals can enhance cellular antioxidant defense, thus boosting protection against age-related diseases [94]. In a molecular setting, polyphenols’ underlying mechanisms are based on neutralizing ROS or upregulating enzymes involved in antioxidant response. 

Higher polyphenol supplementation has been shown to confer a lower risk for several age-related diseases and a positive effect on telomere length dynamics through their antioxidant nature [95]. Green tea [51] and grape seed [96] have been confirmed to elongate telomeres through their antioxidant nature. For example, obese women submitted to green tea administration for a short duration (8 weeks) exhibited telomere length elongation [88,97]. Since telomere shortening results from oxidative stress-mediated damage [88], the protective nature of green tea in obese women seems reasonable owing to its antioxidant nature [88,97].

Another mechanism by which polyphenols regulate telomere length dynamics is through affecting inflammatory responses. A grape component (resveratrol) has drawn particular attention since studies have indicated that it can decrease insulin production. The significant mechanisms underlying resveratrol’s protective nature are based on the upregulation of the anti-inflammatory molecule peroxisome proliferator-activated receptor gamma (PPARγ) and reduction in oxidative stress [98]. Similarly, (epi)gallocatechin gallate triggers programmed cell death and induces the growth of dendritic cells. It also downregulates molecules involved in antigen presentation by dendritic cells [99]. In addition, the combination of epigallocatechin gallate (EGCG), quercetin, and carvedilol might prevent telomere shortening and loss of telomere repeat-binding factor 2 (TRF2) expression, thereby hindering cardiac myocyte death [100]. Regarding the immunosuppressive properties of tea polyphenols, they can decrease Th1 cell development and the number of Th9 and Th17 cells, while resveratrol reduces the number of circulating Th17 cells. These effects have been observed in murine models of rheumatoid arthritis (RA) [101,102].

Polyphenols are also beneficial in orchestrating telomere length dynamics by ensuring genome stability. In grape seeds, procyanidins are oligomers of (epi)catechin, and (epi)catechin gallate units were proved to protect against DNA oxidation by 67% in individuals after their daily intake for 30 days [103]. Accordingly, administering grape seed extracts as sources of polyphenols can reduce genomic instability through telomere length elongation in Alzheimer’s illness animal models [104]. In this regard, telomere shortening and increased serum 8-OHdG levels have been observed in patients with Alzheimer’s disease, suggesting their prognostic value for monitoring the Alzheimer’s disease [105]. In another case, 64 patients with type 2 diabetes (T2D) were randomly assigned to receive hesperidin supplementation and a placebo group was also used [106]. Following the 6-week treatment, the hesperidin treatment group’s serum 8-OHdG concentration was reduced by 23% compared to the placebo group [106]. Apart from hesperidin’s contribution to genome stability, it has been shown that hesperidin has ROS scavenging and anti-inflammatory properties [106].

### 5.2. The Effects of Vitamins and Minerals on Telomere Length Dynamics

Many studies have highlighted the positive effects of vitamins (such as vitamins C, D, and E, folate, and β-carotene) and minerals on the prevention of telomere shortening. Research in this field has shown that the underlying molecular mechanism of vitamins and minerals is based on reducing oxidative stress, fostering innate immune responses, preventing DNA damage and triggering DNA repair processes, controlling the metabolism of glucose and mitochondria, and raising telomerase activity, thus delaying the aging process of cells [107]. Of particular interest, several vitamins serve as substrates or cofactors in DNA replication and repair [108] and in the defense mechanisms involved in eliminating endogenous or exogenous genotoxins [109].

To ensure genome stability, the increased intake of many different micronutrients, such as B-2, B-3, B-6, B-12, folate, C, and E vitamins and zinc, iron, magnesium, calcium, and selenium minerals, has been associated with protection against DNA damage [109,110]. A more complex supplement containing several vitamins (specifically, A, B1, B2, B3, B4, B5, B6, B7, B9, B12, C, D, E, and K) and 18 minerals (including selenium and zinc) has been reported to protect telomeres against damage through reducing micronuclei (MN) formation by 69% [111]. Consistent with the ameliorative effect of vitamins against DNA damage, vitamin B12 has been reported to hinder MN formation in healthy young individuals and to a greater extent in healthy elderly people [84]. In contrast, MN formation was unaffected by vitamin C or E administration [84]. In addition, another RCT proved the antioxidant nature of β-carotene, vitamin C, vitamin E, and selenium together, showing a significant (60%) decrease in chromosome abnormalities (Cas) [112]. Increasing the intake of β-carotene, folic acid, magnesium, and vitamins E and C in a standard diet accounted for telomere length elongation [113]. Meanwhile, a group of people aged 35 to 55 showed a rise in lymphocytic telomerase activity without any change in telomere length after receiving dietary supplements containing a commercial multivitamin preparation containing omega-3-fatty acids, carotenoids, coenzyme Q10, selenium, vitamin D, and alpha-tocopherol for 12 weeks [114]. According to the latter study, antioxidant therapy improved several aging parameters without affecting lymphocyte telomere length [114]. These contradictory findings suggest that telomere shortening is not always linked to aging, implying that aging is a multifaceted process encompassing telomere length and telomerase regulation.

In addition, the relationship between immunosenescence and telomere dysfunction is well-established and particularly interesting in individuals with autoimmune diseases [115]. For example, immunosenescence is linked to telomerase deficiency in RA patients [115]. For this reason, understanding telomerase’s regulation is crucial to developing strategies to reduce immunosenescence, as telomerase is the central controller of telomere length [116].

In this context, vitamins D, B12, and A have been shown to affect many autoimmune diseases positively thanks to their crucial role in immune responses [117,118,119]. Consistent with this, iron, zinc, magnesium, copper, and selenium deficiencies may impair immune function or cause long-term systemic inflammation problems [120]. Since an overdose with mineral supplementation can be accompanied by adverse effects, any mineral supplements should only be taken in the recommended dosages medically [120].

#### 5.2.1. The Effects of Vitamins on Telomere Length Dynamics

Vitamin A use seems to affect telomere length dynamics in an age-dependent manner. In adults, a positive relationship has been observed between the amount of serum vitamin A derivatives (α-carotene, trans-β-carotene, cis-β-carotene, and β-cryptoxanthin) and telomere length [121,122]. In this context, vitamin A administration has been proved to prevent telomere shortening in individuals aged between 19 and 20 [123].

The contribution of vitamin C to telomere length maintenance has also emerged. A relatively recent cross-sectional study using data from the National Health and Nutrition Examination Surveys (NHANES) database has illustrated that telomere length elongation depends on vitamin C intake. Indeed, greater vitamin C administration is correlated with telomere length elongation to a greater extent [124].

Vitamin B and its derivatives play a significant role in sustaining telomere integrity. In particular, telomere length has been positively linked to vitamin B6, vitamin B9 (folate), and vitamin B-12 administration in humans [125,126,127]. For instance, insufficient folate and/or vitamin B-12 can account for defective nucleotide synthesis, thus generating oxidative DNA damage [128,129]. Indeed, it has been documented that vitamin B6 deficiency increases the risk of all-cause mortality in CVD patients. In contrast, patients with high amounts of vitamin B6 are characterized by telomere length elongation through reduction in oxidative stress and inflammation [130]. This research partially sheds light on the possibility that elevated oxidative stress and inflammation could hasten telomere shortening [131].

Folate is essential for maintaining DNA methylation and integrity, affecting telomere length dynamics. It has been documented that telomere length and plasma folate content are interrelated in a sex-independent manner [132,133]. For example, folate deficiency seems to account for telomere shortening through a range of molecular mechanisms, including unsuccessful shelterin protein complex protein attachment to telomeric DNA, increased uracil incorporation into DNA that in turn causes DNA damage, and different epigenetic modifications to DNA excision in telomere structures [82]. In more detail, low folate availability causes uracil replacement of thymidine in telomeres, contributing to the formation of DNA damage in telomeric sequences [134]. In particular, folate deficiency causes uracil misincorporation into DNA and accounts for MN formation and single-strand breaks (SSBs) [134]. Replicative forks are destabilized due to uracil misincorporation, mediating telomere shortening [132,133]. In addition, low folate levels are linked to genomic DNA hypomethylation [135], which affects telomere length in a sex-dependent manner [136]. Furthermore, a polymorphism in the folate pathway, such as the C677T polymorphism of the methylene tetrahydrofolate reductase (MTHFR) gene, is associated with DNA hypomethylation, thus contributing to telomere length elongation [133].

Vitamin B12 has been shown to regulate oxidative stress and methylation levels, affecting genomic integrity and cellular aging. A vitamin B12-dependent process catalyzes the methylation of homocysteine to produce methionine, the precursor of S-adenosyl methionine (SAM). When vitamin B12 levels are insufficient, DNA methylation levels are hindered by lowering the concentration of SAM and increasing the amount of homocysteine [137]. For example, vitamin B12 deficiency has been documented to cause high levels of homocysteine, oxidative stress, and low methylation capacity, contributing to telomere shortening [137]. Regarding the association of vitamin B12 and telomere length, it has been proved that individuals who received multivitamin supplements, including vitamin B12, had longer telomeres due to the antioxidant properties of vitamin B12 [138]. Vitamin B12 is accompanied by a considerable decrease in homocysteine levels and inflammation [131]. Despite its significance in the methylation process, vitamin B12 plasma concentration shows a neutral relationship with telomere length [135]. In a recent RCT, no statistically significant difference pertinent to telomere length was observed during infancy [139]. In particular, vitamin B12 was administered to infants aged 6–11 months daily throughout one year [139]. As a result, the effect of vitamin B12 on aging seems neutral [139].

At the genome-wide association level, a Mendelian randomization (MR) study has underlined that single-nucleotide polymorphisms (SNPs) related to folate and vitamin B12 pathways can reduce susceptibility to vitiligo autoimmune disease [117]. Accordingly, a recent systematic review has proved that high serum vitamin B12 levels are positively implicated in primary cholangitis (PBC) cases, and vitamin deficiency is observed in autoimmune liver disease (AILD) [140].

In addition, vitamin D and vitamin E have been shown to exert a considerable beneficial effect on telomere length [12,141]. Vitamin E (α-tocopherol) has been shown to prevent telomere shortening in an age-dependent manner in human brain microvascular endothelial cells due to its antioxidant properties [142]. Importantly, vitamin D has gained the spotlight because it can reduce susceptibility to aging-associated disorders and all-cause mortality [143]. Numerous studies have been conducted on vitamin D intake and its positive relationship to telomere length [135].

Regarding the antioxidant properties of vitamin D, it has been shown that vitamin D intake protects telomeres from oxidative stress-mediated DNA damage, thereby contributing to the attenuation of telomere shortening [135]. For example, vitamin D can lower 8-OHdG levels in colorectal epithelial crypt cells [82]. Regarding the anti-inflammatory nature of vitamin D, the beneficial impact of vitamin D administration on aging has been proved through the immunosuppressive characteristics of its active form (1α,25 dihydroxyvitamin D3-[25(OH) D3] [135]. Indeed, the inverse correlation between the inflammatory marker C reactive protein (CRP) and plasma levels of vitamin D confirmed the immunoinhibitory role of vitamin D [135]. For example, vitamin D was reported to reduce the expression of IL-2 and IFN-γ [69], thereby compromising telomere shortening [135].

Another mechanism by which vitamin D lowers telomere shortening is the activation of telomerase action. In a double-blind RCT, a higher serum D3 [25(OH) D3] concentration was associated with telomere length maintenance in obese African American volunteers who received vitamin D3 for four months [144]. The higher serum [25(OH) D3] levels of the participants were significantly related to the higher telomerase activity of their PBMCs [144].

In the context of mental disorders, a case–control interventional study has illustrated the positive impact of vitamin D supplementation on telomere length in hemodialysis patients who received calcitriol or analogs for at least six months [145]. A comparative cross-sectional study showed that individuals with successful aging (SA) had considerably higher vitamin D levels than those who experienced mild cognitive impairment (MCI). Additionally, the higher vitamin D levels of the SA group were correlated with longer telomeres compared to those of MCI patients [146].

In the context of autoimmune diseases, low serum dihydroxy vitamin 25(OH) D3 levels have been linked to a higher risk of acquiring several immune-related illnesses [147,148]. In particular, vitamin D elimination has been proven to be related to the development of several autoimmune complications like rheumatoid arthritis (RA), systemic lupus erythematosus (SLE), and Sjogren’s syndrome [149]. For example, Hoffecker et al. showed a significant relationship between serum [25(OH) D3] concentration and telomere length in both healthy individuals and vitamin D-deficient African American Gullah women with systemic lupus erythematosus (SLE) [150]. The statistically significant positive correlation of telomere length with vitamin D levels was determined by using qPCR on extracted telomeric DNA from PBMCs [150]. Meanwhile, patients with SLE also had shorter telomeres after 2.8 years of follow-up than patients whose [25(OH) D3] levels were repleted [150].

In addition, vitamin D insufficiency has also been implicated in the pathogenesis of systemic sclerosis, ankylosing spondylitis, psoriatic arthritis (PsA), rheumatoid arthritis (RA, systemic lupus erythematosus (SLE) and idiopathic inflammatory myopathies [149]. In Sjogren’s syndrome patients, their low vitamin D levels seemed to account for the emergence of neuropathy and lymphoma [149]. As a result, vitamin D supplementation may hinder the pathogenesis underlying autoimmune disease and attenuate pain. In addition, vitamin D insufficiency has been revealed to be correlated with the pathogenesis of autoimmune thyroid disorders, although the underlying mechanisms remain unclear [151]. According to recent research, low vitamin D status has been linked to autoimmune thyroid illnesses like Hashimoto’s thyroiditis and Graves’ disease, and thyroid malignancies have also been linked to compromised vitamin D signaling [152].

Notably, the active component of vitamin D, [25(OH) D3], has been shown to regulate the innate and adaptive immune system and endothelial membrane integrity [147]. It is well-established that [25(OH) D3] has a central role in the function of several immune cell types, such as T, B, macrophage, and dendritic cells. Since the vitamin D receptor (VDR) is sustainably expressed by cells of myeloid origin, it is logical that 25(OH) D3 has a beneficial effect on macrophages and monocytes. In addition, 25(OH) D3 stimulates the growth of monocytes and the generation of IL-1 and the antimicrobial peptide cathelicidin by macrophages, enhancing their function against certain microorganisms. In antigen-presenting cells like dendritic cells, it seems that 25(OH)D3 prevents dendritic cell maturation by hindering the expression of MHC class II, CD40, CD80, and CD86. In T cells, 25(OH) D3 seems to mitigate the function and proliferation of CD4^+^ and CD8^+^ T cells. It decreases the secretion of IL-2, IL-17, and interferon-γ (IFNγ), whereas it boosts the function of forkhead box protein 3 (FOXP3)^+^ regulatory T (Treg) cells and IL-10-producing T regulatory type 1 (TR1) cells [119].

In this context, vitamin A’s metabolite (retinoic acid) is also essential in organizing T and B cell populations in the gut. It induces the differentiation of IgA+ antibody-secreting cells (ASCs) [119]. Since the immunomodulatory role of vitamin A has been established, disturbed intestinal immune responses have been linked with vitamin A insufficiency, which leads to higher morbidity due to gastrointestinal and respiratory infections [119]. In addition to this, retinoic acid has also been demonstrated to induce adaptive immunity by boosting the differentiation of T-helper-2 (TH_2_) cells. In parallel with this, retinoic acid has been reported to hinder the differentiation of T helper 17 (TH_17_) cells. It encourages the formation of T regulatory cells (Treg) through upregulating forkhead box protein 3 (FOXP3)+ and downregulating receptor-related orphan receptor-γt (RORγt) in the presence of transforming growth factor-β (TGFβ) [119]. From a clinical perspective, a recent systematic review has provided evidence that vitamin A administration ameliorated complications of autoimmune diseases through modifying cytokine levels [153].

To sum up, vitamins seem beneficial for telomere dynamics and disease management (Table 2). Additionally, relative telomere length was measured through qPCR, which can determine the average telomere length in a cell population in a high-throughput manner [154]. Telomere length provides an indicative measure of cumulative DNA damage, roughly indicating an individual’s biological aging. However, there is a shortage of information regarding the chromosome-specific measurement of telomere length by metaphase quantitative fluorescence in situ hybridization (q-FISH) [155]. Also, qFISH helps to evaluate critical short telomeres in each chromosome in individual cells, rendering qFISH more reliable and precise than qPCR [156].

**Table 2:** Examples of vitamins’ effects on telomere length.

Vitamin	Effect	Method	Reference
β-carotene (6 mg/day), vitamin C (100 mg/day), vitamin E (100 mg/day), and selenium (50 μg/day) in males with myocardial infarction and male controls	Positive (reduction in DNA damage)	-	[112]
Vitamin B6 (groups: <5.6 μg/L, 5.7–8.9 μg/L, 9.0–14.1 μg/L, >14.2 μg/L) in CVD patients	Positive (telomere length elongation)	qPCR	[130]
Vitamin B12 (groups: <259 pmol/L, 260–472 pmol/L, >473 pmol/L) in individuals with coronary angiography	Positive (telomere length maintenance)	qPCR	[131]
High homocysteine (groups: <9.8 μmol/L, 9.9–12.4 μmol/L, 12.5–15.5 μmol/L, >15.6 μmol/L) in healthy individuals	Negative (telomere shortening)	Southern blot	[132]
Vitamin D (60,000 IU/month [equal to 2000 IU/day]) oral administration in overweight individuals involved in RCT	Positive (telomerase activation)	-	[144]
Vitamins (A, C, and E)	Positive (inhibition of oxidative stress and inflammation)	-	[84]
Vitamin C (groups: <41.5mg/day, 41.5–107.75 mg/day, >107.75 mg/day) in healthy individuals	Positive (telomere length elongation)	-	[124]
α-carotene, β-carotenes (cis and trans), and β-cryptoxanthin in healthy individuals	Positive	qPCR	[121]

Abbreviations: qPCR: quantitative polymerase chain reaction, RCT: randomized controlled trial, CVD: cardiovascular disease. This table is not exhaustive of all studies providing data on the effect of vitamins on telomere length.

#### 5.2.2. The Effects of Minerals on Telomere Length Dynamics

The onset and advancement of age-related human diseases have been associated with aberrant amounts of specific minerals in elderly individuals [157]. Different minerals in the diet have different impacts on telomere length. For example, potassium (K) consumption positively affected telomere length in Korean men and women 50 years of age and older [82]. Accordingly, copper (Cu) considerably impacted telomere length [82]. Elevated calcium levels were related to accelerated telomere shortening and senescence in human fibroblasts [82].

Magnesium maintains the chromosome landscape, since it is required for the activity of endonucleases implicated in base excision repair (BER) of DNA [158]. Many enzymes, including those implicated in DNA replication, DNA repair, and RNA synthesis, require magnesium (Mg) to exert their action [158]. Several studies have also reported that magnesium is required for telomere length maintenance. Indeed, both in vitro and in vivo experiments have proved that magnesium deficiency causes telomere shortening through induction of oxidative stress or inflammation or insufficient DNA replication [135]. In the clinical setting, it has been illustrated that increased dietary magnesium intake positively correlates with telomere length in women [135].

Furthermore, zinc is another mineral that encourages longevity and extends life. Zinc is involved in the following biological processes: protein synthesis, apoptosis, and reduction in oxidative stress and inflammation [135,159].

From a molecular perspective, reverse transcriptases, RNA polymerases, DNA polymerases, and DNA repair enzymes are zinc-dependent in cells requiring zinc as a cofactor [135,160]. For example, the poly(ADP-ribose) polymerase involved in DNA repair at DNA damage sites needs zinc to be activated [135]. Of particular interest is that reverse transcriptase telomerase is activated when there is a greater amount of zinc in culture media [135]. For example, zinc sulfate boosted telomerase activity and facilitated telomere length elongation in human adipose-derived mesenchymal stem cells [82]. The zinc mineral has also been shown to augment telomerase activity and DNA integrity and reduce oxidative stress, contributing to telomere length maintenance [82].

In addition, zinc supplementation can reduce telomere damage in an age-dependent manner, as shown in elderly individuals [82]. For example, dietary zinc deficiency has been linked to oxidative damage, even if a direct role for zinc in eliminating free radicals has not been established [159]. Another example is that when maternal zinc levels are low, chromosomal abnormalities, such as chromosome fusions, result in offspring [161]. One cause of chromosomal fusion is telomere cap loss, which may be caused by zinc deficiency-induced DNA damage [161].

From a clinical perspective, zinc deficiency has been frequently observed in cases of accelerated aging and has been linked to several age-related diseases [162]. Indeed, decreases in intracellular labile zinc concentration and the zinc-binding protein metallothionein in PBMCs have been linked to telomere shortening in elderly people [163]. For this reason, dietary zinc supplementation has emerged as a promising treatment option for various age-related illnesses, such as infectious diseases, neurological disorders, and age-related macular degeneration, by reducing the formation of free radicals and pro-inflammatory mediators [107].

In addition, iron supplements have also been linked to shorter telomeres, in contradiction to the effects of other minerals [135]. Iron’s ability to generate free radicals may cause telomere shortening, as observed in individuals who consume iron supplements [135]. However, telomere length is not inversely correlated with iron intake from food or multivitamins, which may contain less iron than iron supplements [135].

The effect of minerals on telomere length can be modulated in a disease context. Patients with coronary artery disease (CAD) had lower telomerase and telomere length due to lower concentrations of copper and zinc [82]. Lower copper–zinc ratios and greater amounts of copper, zinc, and telomerase were observed in patients with chronic obstructive pulmonary disease [82]. Telomerase activity was elevated in bladder cancer patients with a serum zinc deficit [82]. Treatment with zinc and copper increased telomerase activity in breast cancer cell lines.

Thus, telomere length is affected either positively or negatively depending on the mineral (Table 3).

**Table 3:** Examples of minerals’ effects on telomere length.

Mineral	Effect	Reference
Polymineral complex	Positive (reduction in DNA damage)	[111]
Calcium	Negative (high amounts of calcium related to accelerated telomere shortening)	[82]
Copper and potassium	Positive (telomere length maintenance)	[82]
Zinc	Positive (zinc deficiency related to telomere shortening)	[163]
Zinc	Positive (telomerase activation)	[82]
Magnesium	Positive (magnesium deficiency related to telomere shortening through induction of oxidative stress, inflammation, and insufficient DNA replication)	[135]
Iron	Negative (telomere shortening through induction of oxidative stress)	[135]

This table is not exhaustive of all studies providing data on the effects of minerals on telomere length.

## 6. The Effects of Fatty Acids on Telomere Length Dynamics

The MD restricts amounts of animal-derived proteins, added/refined carbohydrates, and SFAs while promoting the intake of plant-based foods that are abundant sources of unsaturated fats and antioxidants [47]. This healthy lifestyle is characterized by high consumption of dietary sources of unsaturated fats, such as MUFAs and PUFAs [47]. SFAs have been reported to exert detrimental effects on human health [17], whereas unsaturated fats have been shown to lower the risk of both total and cause-specific mortality [47].

Avocados, peanut butter, sesame oil, canola, peanut, safflower, and olive oils are rich sources of MUFAs. The plant-derived MUFAs protect against CVD events [164]. Various mechanisms act synergistically to mitigate the positive effects of MUFAs on CVD, with modifications in lipid levels playing a very minor role [164]. Conversely, soybean, maize, and sunflower oils, as well as various nuts and seeds, tofu, and soybeans, are significant sources of PUFAs.

### 6.1. The Effects of Polyunsaturated Fatty Acids on Telomere Length

The long-chain polyunsaturated fatty acids (LC-PUFAs) can be classified as omega-3 (ω3) or omega-6 (ω6), based on the position of the methyl end group’s first double bond. In particular, PUFAs are a family of lipids that are divided into the following categories: omega-3 (*n*-3), omega-6 (*n*-6), and omega-9 (*n*-9) fatty acids. Among the members of the *n*-3 PUFA family, α-linolenic acid (ALA) is considered a basic fatty acid. Meanwhile, ALA *n*-3 transforms to EPA *n*-3 PUFA and DHA *n*-3 PUFA [165]. PUFAs can be acquired through the diet and the internal synthesis pathway [166]. The principal dietary food sources of *n*-3 PUFAs are abyssal fishes, seaweed, seed oil, and nuts, whereas *n*-6 PUFAs are derived from eggs, meat, fresh seafood, and vegetable oil [166]. A-linolenic acid (ALA) mainly exists in nuts and seeds like walnuts, flaxseed, and oils derived from either soybean or canola. Other *n*-3 PUFAs are eicosapentaenoic acid (EPA) and docosahexaenoic acid (DHA), mainly found in fish and other seafood. Although the body can convert ALA to EPA and DHA, the conversion rates are not very high [167]. PUFAs are essential for human health, since they are involved in many biological processes, including cell signaling, blood pressure control, glucose regulation, nervous system functioning, inflammatory responses, and hematopoiesis [168].

From a clinical perspective, neuron development, synaptic membrane operation, lipid raft organization, memory formation, myelination preservation, photoreceptor biosynthesis and operation, neuroinflammation reduction, and neurological protection are all facilitated by *n*-3 PUFAs in models of neurodegenerative diseases [169]. In addition, *n*-3 PUFAs regulate lipid metabolism and vascular endothelial function and contribute to vasorelaxation in CVD models [170]. The *n*-3 PUFAs have been associated with controlling hyperglycemia, lipids, and lowering hepatic steatosis [171].

Regarding the impact of *n*-3 PUFA administration on telomeres, a longitudinal study by Farzaneh-Far et al. revealed an inverse correlation between blood levels of marine *n*-3 PUFAs and telomere attrition rates [172]. According to another recent cross-sectional study, longer telomeres in males were linked to increased DHA intake and canned tuna consumption [173]. A 13-year longitudinal study on Israeli men found an adverse relationship between all macronutrients and telomere length, with unsaturated fatty acids having the strongest effect [174].

In more detail, PUFA supplementation has been proven to benefit human health by attenuating aging and modulating telomere dynamics. Indeed, PUFA administration was associated with a lower risk of age-related disorders [175,176]. For example, elevated levels of *n*-3 PUFAs in the bloodstream rendered people less susceptible to developing age-related illnesses like cancer and heart disease [177]. Numerous studies have also emphasized the positive effects of *n*-3 PUFA supplementation in sustaining good health and reducing all-cause mortality risk. In particular, *n*-3 PUFA administration reduced the risk of all-cause mortality in patients monitored with 16 years of follow-up [177]. Accordingly, multiple cohort studies have demonstrated the positive impact of *n*-3 PUFAs on telomere length and health status [178].

Multiple examples support the positive effect of PUFA administration on diseases by the regulation of telomere dynamics. Initially, the consumption of marine *n*-3 PUFAs in CAD patients was proven to negatively affect their aging through measurements pertinent to telomere length [172]. In particular, a beneficial effect of *n*-3 PUFA administration on delaying telomere shortening was observed in CAD patients over five years, irrespective of other factors [172]. Accordingly, *n*-3 PUFA supplementation greatly impacted telomere length in CAD patients, contributing to the elongation of telomere length [179]. Furthermore, *n*-3 PUFA administration has shown benefits in chronic kidney disease (CKD) patients, contributing to telomere length maintenance through attenuation of inflammation [180]. In particular, supplementation with a cocktail mix of *n*-3 PUFAs composed of EPA, decosapentaenoic acid (DPA), and DHA reversed telomere shortening in neutrophils derived from CKD patients [180]. Lastly, the consumption of *n*-3 and *n*-6 PUFAs has been reported to positively affect telomere length in elderly and middle-aged healthy, sedentary, overweight, and middle-aged individuals [181]. Indeed, there was an inverse link between *n*-6:*n*-3 PUFA ratio and telomere length, indicating that consuming fewer *n*-6 PUFAs and more *n*-3 PUFAs affected cellular aging [181].

Several RCTs have shown the benefits of *n*-3 PUFAs on telomere length in this field. Initially, a double-blind RCT which included healthy, overweight, sedentary middle-aged, and older participants highlighted the incredible impact of *n*-3 PUFAs on telomere length [181]. The participants were randomly assigned to receive either placebo capsules or 1.25 g/day *n*-3 PUFAs or 2.5 g/day *n*-3 PUFAs for four months. One of the proposed underlying mechanisms mediated by *n*-3 PUFA administration was reduction in oxidative stress by substantial reduction in the amount of F2-isoprostanes [181]. Notably, a lower *n*-6:*n*-3 PUFA ratio was related to higher telomere length in the groups that received *n*-3 PUFAs [181]. Although the disparities among the groups were not statistically significant, a lower *n*-6:*n*-3 PUFA balance was linked to telomere length elongation compared to the telomere shortening observed in the placebo group [181]. In particular, the participants’ telomere lengths increased when the *n*-6:*n*-3 PUFA ratio diminished, confirming the beneficial effect of *n*-3 PUFAs on telomere length elongation [181]. In parallel with this, the authors observed a 15% decrease in urine F2-isoprostanes in the *n*-3 PUFA groups compared to the control group [181]. This could be explained by the demonstrated pro-inflammatory activity of *n*-6 PUFAs, while *n*-3 PUFAs acids have been shown to have anti-inflammatory and antioxidant activities, affecting aging through telomere length regulation [68]. In the same context, another RCT showed that *n*-3 PUFA administration protected sedentary, overweight, and middle-aged participants from accelerated aging due to reduced cortisol, inflammation, and telomerase action, further diminishing susceptibility to depression [182].

In the context of mental disorders, another RCT enrolled elderly individuals with mild cognitive impairment (MCI) who received EPA *n*-3 PUFAs plus DHA *n*-3 PUFAs in different combination patterns or linoleic acid (LA) *n*-6 PUFAs and evaluated their telomere lengths [183]. While the results were not statistically significant, the MCI patients who received LA *n*-6 PUFAs showed accelerated telomere shortening compared to the other groups [183]. Notably, the *n*-6 PUFA group presented more intense telomere shortening than the *n*-3 PUFA group. Notably, the MCI patients who received DHA *n*-3 PUFAs presented lower rates of telomere shortening [183]. In another study, 33 Australians over 65 with MCI participated in a randomized parallel-group pilot trial, in which three different administrations containing either *n*-3 PUFAs or *n*-6 PUFAs were given for six months. When telomere length was measured in the participants’ whole blood, those who received *n*-3 PUFAs showed telomere shortening but those who received *n*-6 PUFAs did not show any difference in their telomere lengths [183]. In line with the above, several observational studies have also reported a relationship between dietary *n*-3 PUFA administration and cognitive performance in healthy and elderly individuals, suggesting the regulation of mental function by *n*-3 PUFAs [184]. Indeed, several recent prospective studies and RCTs have also underlined the benefits of *n*-3 PUFA supplementation with respect to mood, depressive symptoms, and cognition [185,186,187]. However, a recent large-scale clinical study [VITAL-DEP (Vitamin D and Omega-3 Trial-Depression Endpoint Prevention)] demonstrated that *n*-3 PUFA administration was not effective in hindering depression in adults [188]. Instead, it raised individuals’ likelihood of depression or clinically relevant depressive symptoms [188]. The VITAL-DEP trial’s final result was consistent with other earlier clinical trials and investigations showing null results for *n*-3 PUFA supplementation in adults with or without memory problems and functional physiological decline [189,190,191].

From a molecular perspective, numerous studies have demonstrated the ability of PUFAs to prevent either oxidative stress or inflammation or apoptosis and their endothelial vasodilator properties, lowering the incidence of age-related abnormalities [192]. For example, a diet enriched in marine *n*-3 PUFAs has been shown to confer antioxidant and anti-inflammatory properties through increasing the action of antioxidant enzymes like glutathione peroxidase (GPx), catalase (CAT), and superoxide dismutase (SOD) and anti-inflammatory markers [193,194], thereby attenuating oxidative DNA damage and preventing telomere erosion. The mechanisms underlying the protective effect of *n*-3 PUFAs on aging were attributed to the upregulation of transcription factors, such as sterol-regulatory element-binding proteins (SREBPs) and peroxisome proliferator-activated receptors (PPARs) [195,196]. All these molecular mechanisms demonstrated in animal and human studies have highlighted the involvement of *n*-3 PUFAs, particularly EPA and DHA, in reducing inflammatory and tissue oxidation markers contributing to autoimmune disease [197]. In particular, EPA, DHA, and DPA can restore immune system health in the early stages of the autoimmunity process [198]. As a result, the impact of *n*-3 PUFAs on human aging remains obscure due to the heterogeneity of the data. For this reason, it is imperative to proceed to the careful design of clinical trials that will include all the factors related to *n*-3 PUFAs in humans, especially in the elderly population.

### 6.2. The Effects of Monounsaturated Fatty Acids on Telomere Length

The main bioactive elements of olive oil are virgin olive oil (VOO) and extra virgin olive oil (EVOO), which are linked to various health benefits [199]. EVOO is an MD staple known for its outstanding nutritional properties. EVOO exerts its impact on human health through its fatty acid composition and bioactive elements like phenolic compounds [199]. Lozano-Castellón et al. used a method that relied on UHPLC combined with electrospray ionization and MS/MS (UHPLC-ESI-MS/MS) for the identification of the four significant secoiridoids, which correspond to the essential phenolic compounds in EVOO [200].

From a molecular perspective, the MD seems beneficial for telomere length dynamics by targeting oxidative stress, low-grade inflammation, and DNA damage, thereby preventing telomere shortening [201]. The MD dietary strategy enriched with olive oil can attenuate the onset and the progression of age-related diseases through the downregulation of ROS accumulation and pro-inflammatory mediators [201]. In this context, olive oil can increase antioxidant defense through upregulating catalase enzymes in aged cells, conferring protection against disease progression [202]. The antioxidant properties of olive oil components have also been confirmed through HO-1 induction and increased action of the Nrf2 transcription factor, thus blocking senescence [203]. Additionally, HO-1 is known for its anti-apoptotic, antioxidant, and anti-inflammatory nature [201]. Last, phenolic compounds found in olive oil can prevent DNA damage by safeguarding APEX1, a DNA repair gene crucial for lowering the risk of age-related illnesses [204].

In one RCT, elderly participants were enrolled to follow three dietary patterns for four weeks. The diet types were distinct due to their enrichment in SFAs, low abundance of fat and high abundance of carbohydrates, and the prevalence of MUFAs involved in the MD dietary plan [205]. After isolating umbilical endothelial cells from patients and culturing them in their sera, the patients’ nutritional habits were associated with their telomere lengths [205]. The results showed that participants who followed the olive oil-enriched MD diet preserved their telomere lengths compared to the patients who followed the other diets, due to reduced oxidative bursts and cell death [205]. In summary, the *n*-3 PUFAs and MUFAs seem beneficial for telomere length homeostasis, as shown in Table 4.

**Table 4:** Examples of fatty acids’ effects on telomere length.

Fatty Acid	Effect	Method	Reference
MD supplemented with EVOO	Positive (reduction in inflammation)	qPCR	[78]
Administration of *n*-3 PUFAs, namely, EPA, DPA, and DHA, in CKD patients	Positive (reduction in inflammation)	qPCR	[180]
Administration of 1.25g/day *n*-3 PUFAs or 2.5 g/day *n*-3 PUFAs	Positive (reduction in oxidative stress)	qPCR	[181]

Abbreviations: MD: Mediterranean diet; EVOO: extra virgin olive oil; PUFAs: polyunsaturated fatty acids; *n*-3: omega-3, EPA: eicosapentaenoic acid; DHA: docosahexaenoic acid; DPA: decosapentaenoic acid; CKD: chronic kidney disease; qPCR: quantitative polymerase chain reaction. This table is not exhaustive of all studies providing data on the effect of fatty acids on telomere length.

## 7. Limitations

Over the years, increasing evidence has substantiated that telomere length can be a marker of biological aging [206]. Nonetheless, epigenetic modifications can also constitute other indicators of health and biological aging related to diseases [206]. Telomere length heterogeneity across tissue and cell types is considered an additional factor in telomere homeostasis [207]. Another limitation is that telomere dynamics can be confounded by other environmental triggers like gender, ethnicity, and disease states [12]. Depending on individuals’ lifestyles, telomere attrition is crucial for monitoring aging; no studies have evaluated the half-lives of lymphocytes and stem cell turnover as alternative indicators of aging [2]. It is unclear whether the MD’s protective benefits with respect to telomere length are due to any of these components alone or to a combination. The simultaneous presence of many antioxidants in the MD can complicate understanding the MD’s benefits against diseases. In addition, there is a lack of precise and clear clinical evidence that MD constituents have a beneficial effect on telomere length and the management of diseases. Some proposed mechanisms underlying the protective impact of the MD have been reported, but more studies are needed to confirm them.

Some limitations of this review include its narrative and non-systematic manner. Moreover, significant heterogeneity among the included studies due to their different study designs precludes the safe extrapolation of conclusions. Additionally, no meta-analysis could be performed to evaluate a net effect.

## 8. Conclusions

One reliable indicator of aging is telomere dynamics, and the MD dietary strategy can positively affect the telomere attrition rate. Since the MD is a positive regulator of telomere length maintenance, numerous clinical data indicate the protective effects of the MD dietary strategy in terms of lowering the risk of age-related diseases. Indeed, much evidence supports the association of polyphenols, fatty acids, vitamins, and minerals with telomere length homeostasis. In a molecular setting, the mechanisms of the MD’s underlying ameliorative effects on age-related disorders can be attributed to the reduction in inflammation, oxidative stress, and mitochondrial dysfunction and the activation of telomerase action, thus exerting a significant impact on telomere length dynamics. To sum up, comprehensive RCTs are essential to alleviating the risk of several age-related diseases. The correct design of RCTs can allow MD components to be effective, safe, and well-tolerated under age-challenged conditions. Lastly, guidelines should be based on drug–food interactions to avoid significant pharmacokinetic or pharmacodynamic interactions and increase the synergistic effect of the molecular mechanisms in maintaining telomere integrity.
